# Supplementary figures and images for: Gamete Collection, Artificial Fertilization and Captive-Rearing of Eggs in a Terrestrial-Breeding Anuran with Parental Care: Alytes obstetricans
Source: Animals (Basel). 2023 Sep 4;13(17):2802. doi: 10.3390/ani13172802 (PMC10487003; doi:10.3390/ani13172802)

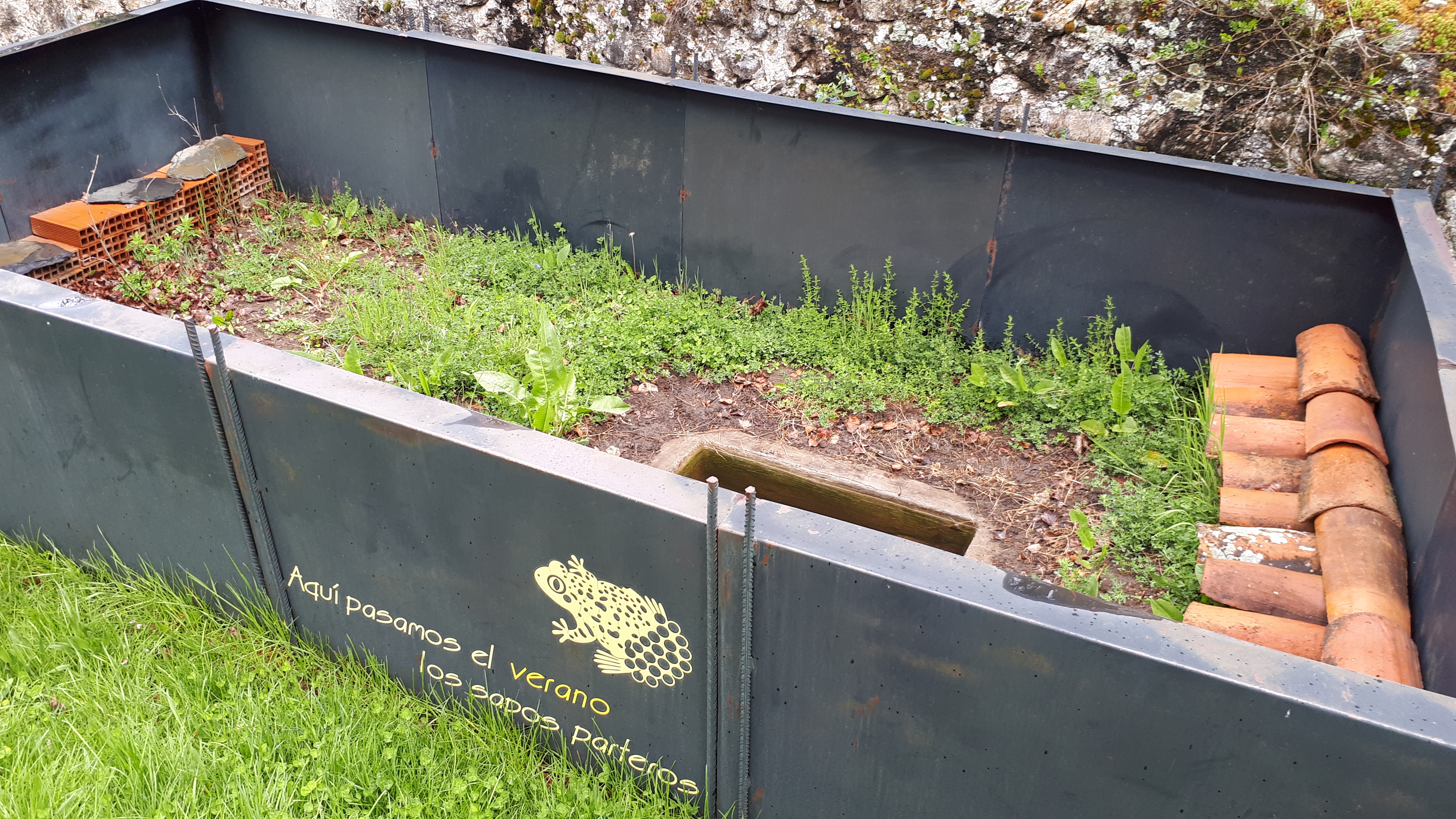

Supplement: Supplementary file 1 [file animals-13-02802-s001.zip › animals-2559850-supplementary.jpg]
